# End-of-life care in children with hematologic malignancies

**DOI:** 10.18632/oncotarget.21188

**Published:** 2017-09-23

**Authors:** Jessica I. Hoell, Jens Warfsmann, Stefan Balzer, Arndt Borkhardt, Gisela Janssen, Michaela Kuhlen

**Affiliations:** ^1^ Department of Pediatric Oncology, Hematology and Clinical Immunology, Center for Child and Adolescent Health, Medical Faculty, Heinrich Heine University of Dusseldorf, Dusseldorf, Germany

**Keywords:** palliative care, children, pediatric, home care, hematologic malignancies

## Abstract

**Introduction:**

Hematologic malignancies (HM) represent the most common neoplasms in childhood. Despite improved overall survival rates, they are still a major contributor to cancer death in children.

**Aims:**

To determine the proportion of children with HM in pediatric palliative care (PPC) and to identify the clinical characteristics and symptoms in comparison to children with extracranial solid tumors (non HM patients).

**Patients and Methods:**

This study was conducted as a single-center retrospective cohort study of patients in the care of a large specialized PPC team.

**Results:**

Fifteen HM and 50 non HM patients were included. Symptoms in which HM patients scored significantly higher than non HM patients were mucositis, difficulty moving, somnolence, fatigue, petechiae and paleness. Blood transfusions were more frequently administered to HM patients, but large external hemorrhage was not observed in any child. A large variety of drugs and appliances were needed by the patients, with morphine being the most frequently prescribed drug. During the study period, a much larger and over the years even increasing number of HM patients (not in the care of the PPC team) died in hospital with an (assumed) curative intent, with two thirds dying in the ICU.

**Conclusions:**

Children with HM were referred to outpatient PPC with almost the full clinical picture of advanced leukemia. Noteworthy, the number of children with HM dying at home is decreasing in our center, instead a substantial proportion received high-intensity medical hospital care including novel anticancer therapies. These patients thus seem to be at an increased risk of dying in hospital as the right time to transfer them to palliative care is oftentimes missed.

## INTRODUCTION

Hematologic malignancies (HMs) are the most common neoplasms in childhood representing about 45% (30.6% leukemias, 14.2% lymphomas) of all newly diagnosed pediatric cancers [1]. In Germany, approximately 800-900 children and adolescents are diagnosed with a HM per year. Despite increasing intensification of multi-modal therapy and significantly improved overall survival rates, given the relative frequency of the diagnosis, still approximately 100-150 (about 15%) of them will ultimately die every year from their disease.

Previous research has demonstrated that despite continuous improvement in palliative care children dying of cancer still substantially suffer and that the requirements of these children strongly depend on tumor entity and localization [2–6].

According to a retrospective Swedish record analysis, children with HM dying in hospital were more frequently treated with curative intended therapy including chemotherapy, transfusions, and antibiotics closer to death and, thus, were less likely to be recognized being beyond cure and referred to palliative care teams (PCTs) as compared to central nervous system and extracranial solid tumors [7, 8]. However, little is known about the distinct requirements of these children at the end of life – especially in the light of home-care -, as a much larger body of publications deals with the requirements of children with CNS and solid tumors. Hence, practice of palliative home-care of children with HM is so far adapted from hospital care and/or best practice delivered to children with other tumor entities. One might hypothesize that - despite the home setting - some of these children are treated more intensively than would be required by the actual clinical symptoms. For example, regular transfusions might be administered depending on blood levels instead of bleeding signs in a situation, in which many parents might fear external hemorrhage.

The aim of the study was - first - to determine the proportion of children with a hematologic malignancy in pediatric palliative home care in relation to the number of children dying of a HM in hospital, - second - to determine the clinical characteristics and symptoms, nursing requirements, and time of referral to PCT, - third - to compare these data with data of children with extracranial solid tumors.

## RESULTS

### Only the minority of patients with hematologic malignancies are referred to palliative care and numbers are declining over recent years

In the study period a total of 335 children, adolescents and young adults (subsequently referred to as “children” or “patients”) were cared for by the PCT at home. In the study period, the PCT looked after 123 children with cancer, of which 15 (12%) children were diagnosed with a hematologic malignancy. Of those, 8 (53%) patients presented with acute lymphoblastic leukemia (ALL), 3 (20%) with acute myeloid leukemia (AML), 3 (20%) with non-Hodgkin lymphoma (NHL), and 1 (6%) with Hodgkin lymphoma (HL). Ten patients (67%) underwent hematopoietic stem cell transplantation (SCT). Except for one AML patient, who presented with refractory disease in the firstline treatment protocol, and one lymphoma patient, who progressed under therapy, all other patients had relapsed with their disease at least once, nine patients twice or more than that. Eight of those patients previously underwent hematopoietic stem cell transplantation (SCT). Only one patient had two relapses following SCT, all other patients were referred to palliative care following their relapse after SCT. Median time from the primary diagnosis to referral to palliative care was 1.3 years (range 0.2-6.7). Demographic characteristics and general information on palliative home care are given in Table 1.

**Table 1:** Demographic characteristics and general information on palliative home care in children with hematologic malignancies (n=15) and extracranial solid tumors (n=50)

No.	HM	non HM	*p-value*
	15	50	
Diagnoses	ALL 8	Ewing tumor 11	
	AML 3	NBL 9	
	NHL 3	RMS 9	
	HL 1	OS 6	
	Other 0	Other 25	
Gender, male n (%)	11 (73.3%)	26 (52.0%)	*0.23*
Age at referral, median(range in years)	12.2 (2.1-22.4)	15.1 (1.5-28.3)	*0.43*
Duration of palliative care, median (range in days)	12.3 (6-122)	41 (2-502)	*0.07*
Home visits, median(range in no.)	5 (1-14)	7 (1-68)	*0.23*
Rehospitalization, n	0	6	*0.32*
Deceased, n (%)	15 (100%)	50 (100%)	
Place of death, n (%)			
At home	14 (93.3%)	47 (94.0%)	*0.66*
In hospice / pall. unit	1 (6.7%)	1 (2%)	*0.55*
In hospital	0	1 (2%)	*1.0*
Age at death, median(range in years)	12.2 (2.1-22.4)	15.2 (1.5-28.3)	*0.27*

**Figure 1:**
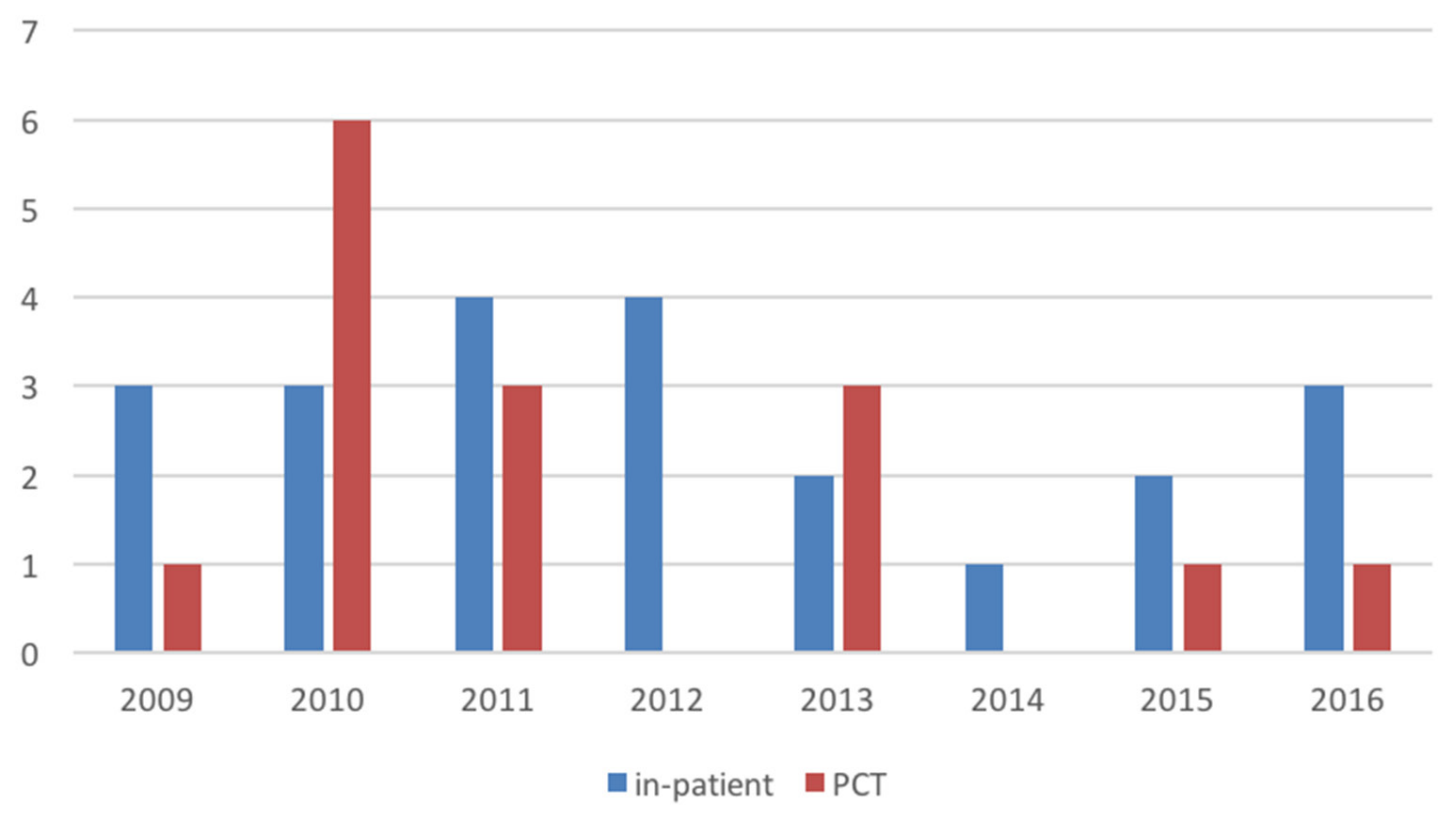
Overview of the HM patients dying in a hospital setting (including ICU) not cared for by the PCT (blue) and those HM patients seen by the PCT (red) Numbers of patients are given on the y-axis, analyzed years on the x-axis.

The number of children with HM cared for in the home setting was likely lower than expected because of an increasing number of children with relapsed or refractory leukemia undergoing intensive relapse treatment including a second SCT and often then dies during intensive chemotherapy/SCT while still being cared for in hospital. This is reflected by the higher number of 22 comparable patients who died in our hospital during the study period, with 14 of them dying in an intensive care setting. Comparing the patients dying (with assumed curative intent) in the hospital to those under the care of the PCT by year, one notices a downward trend of those patients referred to PCT from 2009 until 2016 (Figure 1).

We then decided to compare the HM patients to those diagnosed with extracranial tumors (non HM patients). In the same study period, the PCT took care of 50 non HM patients. The most common diagnoses were Ewing tumors (n=11, 25%), neuroblastomas (n=9, 15%), rhabdomyosarcomas (n=9, 15%) and osteosarcomas (n=6, 10%). The other 25 patients suffered from rarer tumor entities such as desmoid small round cell tumor, hepatocellular carcinoma, cholangiocellular carcinoma and synovial sarcoma. For demographic data in comparison to children with HM also see Table 1. During the study period, only one comparable non HM patient died in the hospital.

Time from start of palliative home care to death was 12.3 days in care (median) in children with HM compared to 41.0 in non HM (*p=0.07*), although the range of days in palliative care was larger in children with non HM (2 to 502 days) compared to children with HM (6 to 122 days). There was no significant difference between the number of home visits (6.1 vs. 9.4 visits (mean)) between both groups. In both patient groups, most patients died at home or in hospice.

One HM patient died of an acute event, most likely intracranial hemorrhage, the other patients died of disease progression. Two HM patients (unstoppable vomiting, large mediastinal tumor, respectively) received palliative sedation.

### Patients with hematologic malignancies present with symptoms of very advanced disease when admitted to pediatric palliative care

Children with HM were seen 3.1 times on average in the first seven days of care (HM patients: mean 3.1 / median 3 visits; non HM patients: mean 2.2/ median 2 visits (*p=0.05*)). With each recorded symptom counted on each visit within the first 7 days of palliative care, the median number of symptoms per HM patient amounted to 94 (range 12-190), indicating that these patients have a very high symptom burden when being admitted to pediatric palliative care (PPC). The median number of recorded complaints within the first 7 days of PPC by children with non HM was 47 (0-236) and, thus, clearly lower compared to that of HM children (*p=0.01*).

**Figure 2:**
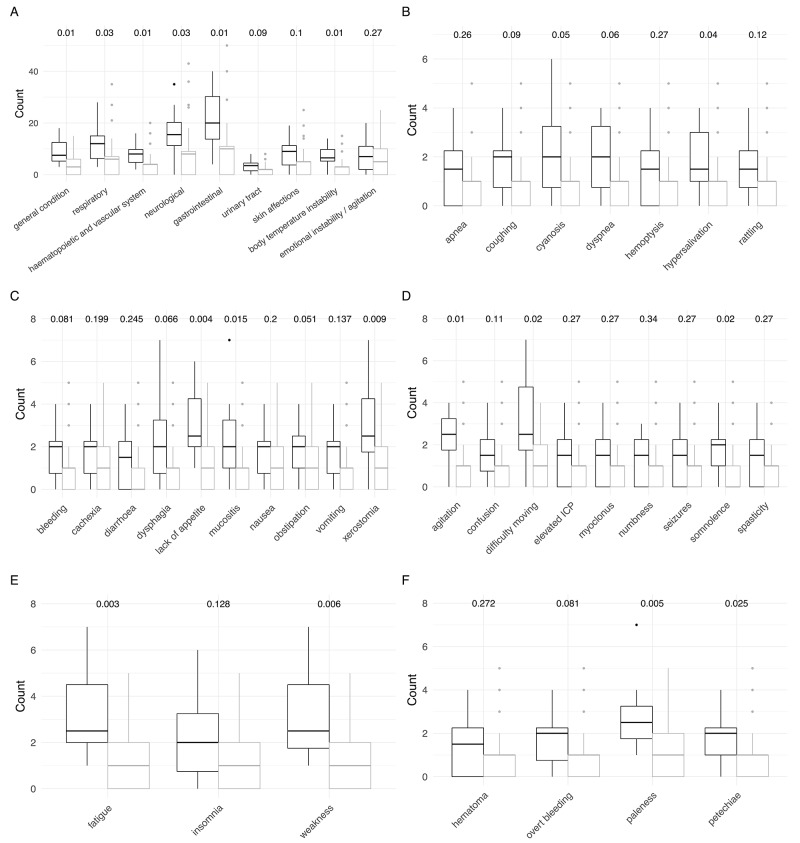
Comparison of signs and symptoms of children with hematologic malignancies (black) and extracranial solid tumors (grey) at referral (first 7 days of care) P-values obtained from Wilcoxon rank sum tests with continuity correction are shown on top. **(A)** Detailed overall signs and symptoms. **(B)** Detailed respiratory symptoms. **(C)** Detailed gastrointestinal symptoms. **(D)** Detailed neurological symptoms. **(E)** Detailed symptoms in the category general condition. **(F)** Detailed hematopoietic and vascular symptoms.

Patients with HM presented with a variety of symptoms, which differed from the presenting symptoms of non HM patients (Figure 2). Children with HM scored significantly higher in the following symptom categories: general condition (*p=0.01*), hematopoietic/vascular system (*p=0.01*), body temperature instability (*p=0.01*), respiratory (*p=0.03*), neurological (*p=0.03*) and gastrointestinal (*p=0.04*). Looking into more detail into the different categories, the specific complaints / symptoms were lack of appetite (*p=0.02*), mucositis (*p=0.02*), dry mouth (*p=0.01*), difficulty moving (*p=0.02*), somnolence (*p=0.02*), fatigue (*p=0.003*), weakness (*p=0.01*), petechiae (*p=0.02*) and paleness (*p=0.005*). Taken together, HM patients suffered from signs and symptoms of advanced leukemia.

The most frequently reported symptoms in patients with non HM were neurological and gastrointestinal problems as well as emotional instability.

**Figure 3:**
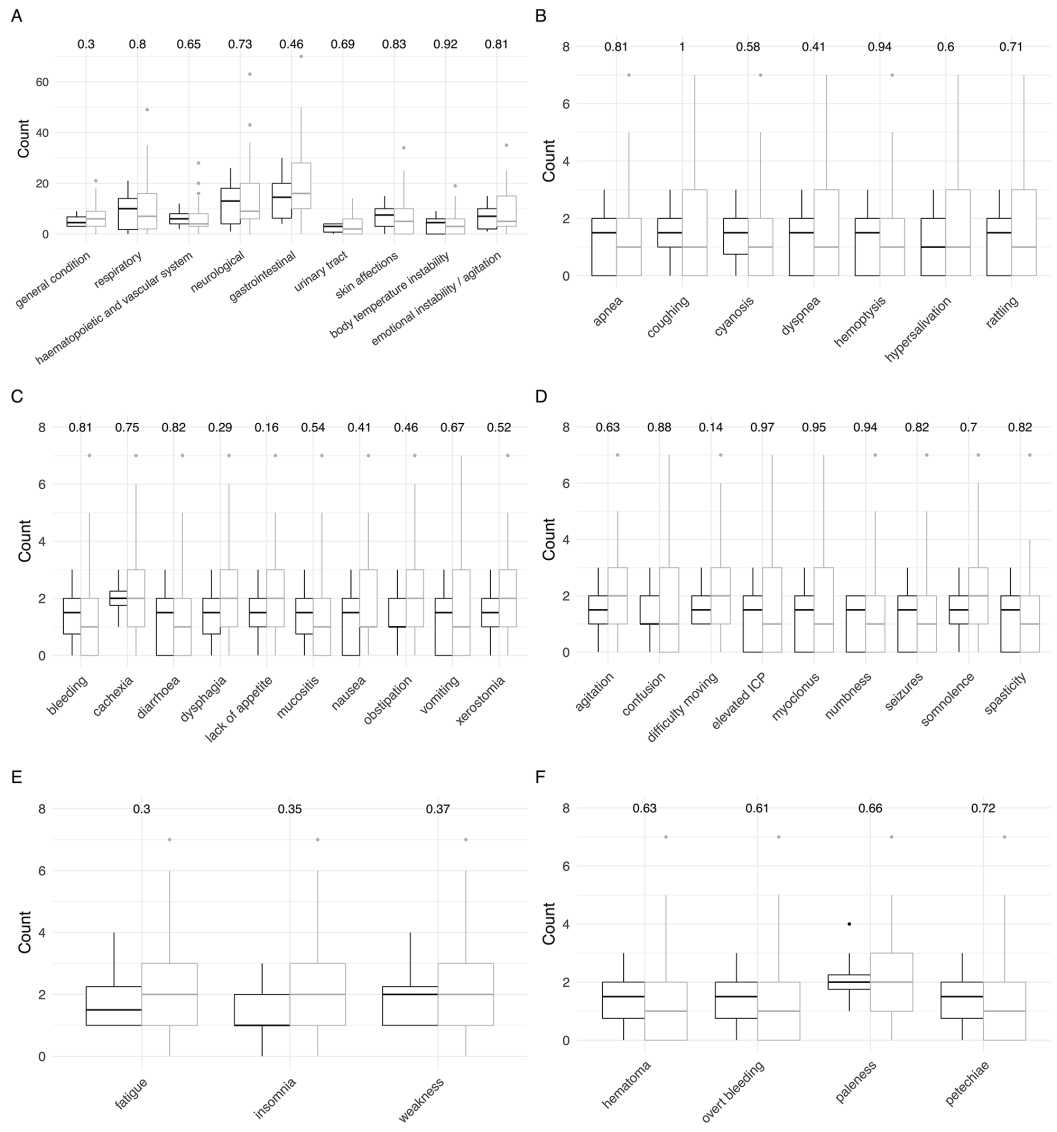
Comparison of signs and symptoms of children with hematologic malignancies (black) and extracranial solid tumors (grey) at the end of care (last 7 days) P-values obtained from Wilcoxon rank sum tests with continuity correction are shown on top. **(A)** Detailed overall signs and symptoms. **(B)** Detailed respiratory symptoms. **(C)** Detailed gastrointestinal symptoms. **(D)** Detailed neurological symptoms. **(E)** Detailed symptoms in the category general condition. **(F)** Detailed hematopoietic and vascular symptoms.

Notably, looking at the symptoms recorded during the last seven days of care, all differences disappeared between the two patient groups, not a single category reached statistical significance (Figure 3). When again counting each recorded symptom on each visit within the last 7 days of palliative care, the median number of symptoms per HM patient amounted to 70 (17-141), whereas the number was 50 (0-333) for non HM patients (n.s.).

### (External) Bleeding is not a recurring clinical problem

Mucosal bleeding, hematoma and/or petechiae were recurring problems in children with leukemia requiring platelet transfusions in the home setting, however, not a single child developed massive external hemorrhage, as might have been feared by the parents.

HM patients received a total of 23 transfusions (mean of 1.5 per patient), breaking up into 16 platelet concentrates (7 patients) and 7 packed red blood cells (5 patients). In comparison, transfusions in non HM patients totaled up to 50 (mean 1.0 per patient) with 27 platelet concentrates (9 patients) and 23 packed red blood cells (9 patients). Looking into more detail into which non HM patient groups received the transfusions, revealed that 68% (n=34) of all transfusions were administered to the nine neuroblastoma patients, meaning that only 16 transfusions were administered in the remaining 40 patients (mean 0.4 per patient).

Within the last week of life, five transfusions were given each in HM and in non HM patients.

### A large variety of drugs and care tools are needed by the patients and their families

**Figure 4:**
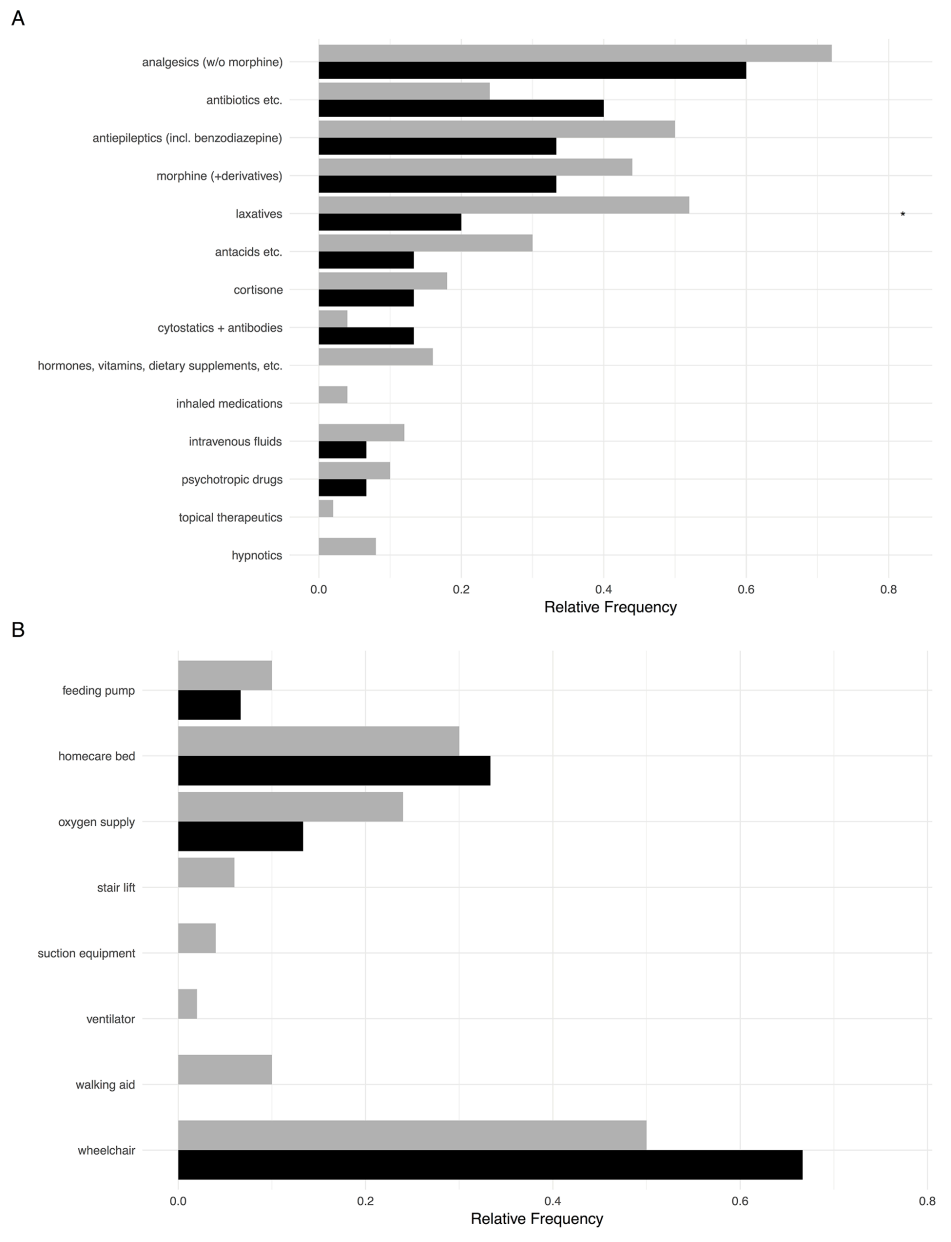
**(A)** Administered treatment including chemotherapy in HM patients (black) compared to children with extracranial solid tumors (grey). **(B)** Medical devices and equipment available to children with HM and non HM.

The median number of administered drugs in HM patients was three (range from 0 to 15). In non HM patients, this number of different drugs was slightly higher (median 4, range 0-18) (Figure 4A). Both the types of medications as well as the number of administered drugs (see below) were similar in both patient groups.

Given the symptoms, the most prevalent drugs included pain medication (including morphine), laxatives and antiepileptics. The latter category also contained benzodiazepines, thus representing more anxiolytics in these patient groups then classical antiepileptics. Cortisone was also frequently prescribed. Rather surprisingly, antibiotics/antifungal medications were also taken by around half the patients in both groups, although they were only administered intravenously in two children. The most common orally taken drugs were trimethoprim/sulfamethoxazole (7x), diflucane (2x), and cefpodoxime (2x). In nine children with HM and in 33 non HM patients, morphine-based analgesics were given via patient-controlled analgesia pumps. Orally prescribed morphine and derivatives are depicted in Figure 4A. Five HM patients and 12 non HM patients were continued on palliative chemotherapy at referral to PPC.

In addition to the different medications, a large variety of medical and therapeutic appliances (Figure 4B) were also needed by the patients and their families with a median number of 1 (range 0 to 5) in HM patients and a median number of 1 in non HM patients (range 0 to 8). Due to impaired mobility, homecare beds and wheelchairs were frequently needed by the families.

## DISCUSSION

Our study shows, that children with HM present with the clinical picture of advanced leukemia at admission to PPC. With transfusions given only based on bleeding signs instead of blood levels no child developed massive external bleeding. The end-of-life phase of HM and non HM patients was mainly characterized by symptoms of disease progression without significant clinical differences. Notably, during the respective time period, one and a half as many children with HM died in hospital whereas only a single child with non HM.

The most frequently reported symptoms in children diagnosed with cancer in general are pain, fatigue, poor appetite, dyspnea, and neurologic symptoms (the latter mainly in children with central nervous system tumors). [2–6, 9–20] Naturally, the underlying malignancy significantly influences the prevalence of some symptoms. [5, 21, 22] Thus, not surprisingly, symptoms of children with HM initially significantly differed from that of non HM patients. At referral to PPC, most children with HM presented with the full clinical picture of advanced leukemia. Although not statistically significant, that led to a higher number of transfusions per child in children with HM compared to non HM children. Noteworthy, most transfusions in non HM children were given to patients with neuroblastoma. Indication to give a transfusion was based on clinical decision, e.g. bleeding signs and weakness due to anemia, instead of laboratory findings. Under this regime, none of the children developed massive external hemorrhage. Interestingly, we found no difference in the frequency of pain – neither on admission to PPC nor at the end of life – between both groups. This is likewise reflected by the similar proportion of patients receiving morphine-based drugs - although not compared by dosages - and the use of patient controlled analgesia pumps.

As is depicted by the relative frequency of symptoms in the last seven days of life, many of the symptoms remained – to a certain extent – unresolved. This is in line with previous reports that relief of symptoms cannot be achieved in a substantial proportion of children dying of cancer. [3, 9, 14, 20]

As was also the case in our study, cancer patients often experience a large number of symptoms that frequently appear together. Therefore, in adult palliative care, tools such as the Edmonton Symptom Assessment System (ESAS) have been developed to identify recurrent symptom clusters among cancer patients and, thus, to improve our knowledge about such symptom clusters and their clinical implications on symptom management and quality of life. [23–26] However, so far, these tools have not been evaluated in PPC and future research is needed to evaluate whether these clusters hold true for pediatric cancer patients.

In addition, symptom assessment in PPC can be difficult due to various reasons. First, child self-reports would be desirable but no widely accepted tool is yet available. Second, symptom assessment scales for physician use such as the Memorial Symptom Assessment Scale (MSAS) are reliable and valid instruments for the assessment of symptom prevalence, characteristics and distress in adults. [27] However, validation of this tool in children with cancer has yielded inconclusive results. [28, 29] As the database used by our PPC team does not (yet) enable documentation of symptoms according to the MSAS, we here report symptom counts as detailed above.

In contrast to the Swedish study reporting on children with HM dying in hospital, in our cohort children with HM were equally frequently treated with antibiotics compared to extracranial solid tumors. [7, 8] This is somewhat surprising as one would expect that children with HM are at higher risk to develop infectious complications and, thus, are more frequently treated with antibiotics.

At referral to PPC, 20-35% of the children in both groups received cancer-directed therapy. This is lower compared to previous reports, in which more than half of the children received cancer-directed therapy in the end of life phase. [3, 9, 20, 30] As some studies indicate that this was in hindsight negatively rated by the parents, discontinuation of cancer-directed therapy on admission to PPC should be discussed with all parents.

Beyond that, treatment and nursing requirements in HM children were comparable to those of children with solid tumors. This reflects that the end-of-life phase of all oncological patients is mostly characterized by steady physical decline and less by disease-specific signs and symptoms. Most children died of disease progression and not of acute complications. [30]

Although this study represents a single center analysis, it highlights a notable downward trend of children with HM being cared for by the PCT and dying at home. Indeed, a substantial number (22) of HM children died in hospital or even in an intensive care unit (14). This confirms the just recently published data from Ontario/Canada (referring to data between the years 2000 and 2012), which demonstrated that children with cancer often received high-intensity medical care at the end of life and that particularly children with hematologic malignancies were at the highest risk to receive the most invasive end-of-life care. [31] It might be hypothesized that this is due to the prognostic uncertainty and concerns about taking away hope and the right time to refer to palliative care in the light of an increasing availability of innovative strategies especially in the field of post SCT relapse. These novel strategies include chimeric antigen receptor T cells and CIK cells.

To put the findings of our study into clinical practice, we have started implementing joint discussions with the treating pediatric oncologist and a member of the PPC when informing the parents about potential novel therapeutic strategies available to their children.

Our hospital is a large tertiary center with a strong focus on the treatment of relapsed and refractory hematologic malignancies offering a large variety of the mentioned experimental therapies. Many patients are referred to receive SCT or when established treatment options have failed and the parents / families wish further innovative therapies. Thus, this might constitute a considerable sampling bias concerning limitation of treatment and transfer to PPC.

In summary, children with hematologic malignancies are referred to outpatient palliative care with almost the full clinical picture of advanced leukemia. Noteworthy, the number of children with HM dying at home is decreasing, instead a substantial proportion of deceased children received high-intensity medical care including novel anticancer treatment strategies. This highlights that, despite an increasing acceptance of palliative care approaches in pediatrics, including pediatric oncology, novel treatment strategies / phase I and II treatments in pediatric oncology may have the risk to miss the point of no return in these children. These patients thus seem to be at an increased risk of dying in hospital as the right time to transfer them to palliative care is oftentimes missed. We believe that within pediatric oncology, there seems to be an increasing need to discuss and question these innovations in order to gain a more comprehensive view.

## MATERIALS AND METHODS

This study was conducted as retrospective single center cohort study at Children´s University Hospital, Dusseldorf, Germany, between 2008/09/01 and 2016/11/15.

Patients were included who fulfilled the following criteria: previous anticancer treatment, diagnosis of an incurable, progressive malignancy, and continuous home care (until death) provided by the specialized pediatric PCT of this institution. Time under investigation was defined as time between start of palliative home care and death or the date of this analysis (November 15^th^ 2016), respectively.

The specialized pediatric PCT is composed of pediatricians and nursing staff with additional training in PPC as well as a social worker. On demand, psychologists are available for crisis intervention. Home visits are conducted at referral to palliative care and thenceforward on a regular basis. The PCT provides a 24 hours/7 days on-call duty by a physician and nursing staff.

Patient data were extracted from two databases (2008/09/01-2012/12/31 and 2013/01/01-2016/11/15), into which the data were routinely entered by the members of the PCT after each patient contact. Information included demographic data, diagnoses, contacts, symptoms, medications, rehospitalization, and place of death.

Symptoms were entered into the web interface in a standardized manner with the entire list of symptoms being completed after each home visit by a physician. To estimate the symptom burden at referral to palliative care and at the end of care, the median number of symptoms was calculated by counting the number of symptoms documented on each home visit within the first and last 7 days of care. For reasons of clarity and better comparability, symptoms were classified into nine categories (general condition, respiratory, hematopoietic and vascular system, neurological, gastrointestinal, urinary tract, skin affections, body temperature, and emotional). The five categories with the highest median number of symptoms (respiratory, gastrointestinal, neurological, general condition, and hematopoietic and vascular symptoms) were further analyzed.

To evaluate the impact of the HM on symptom pattern, requirements, and home care of these children, testing against children with extracranial solid tumors was performed. Hence, all children with extracranial solid tumors cared for by the PCT in the home setting in the corresponding time intervals were additionally analyzed.

Frequency data between both groups (HM/non HM) were compared applying Fisher's exact test (no adjustment for multiple testing). Further, we conducted significance tests on count distributions applying the Wilcoxon rank sum test with continuity correction. All statistical analyses were performed using R (R Core Team 2015).

The study was approved by the local ethics committee (study numbers 4969 and 4461).
